# Circular RNA circSDHC serves as a sponge for miR-127-3p to promote the proliferation and metastasis of renal cell carcinoma via the CDKN3/E2F1 axis

**DOI:** 10.1186/s12943-021-01314-w

**Published:** 2021-01-20

**Authors:** Junjie Cen, Yanping Liang, Yong Huang, Yihui Pan, Guannan Shu, Zhousan Zheng, Xiaozhong Liao, Mi Zhou, Danlei Chen, Yong Fang, Wei Chen, Junhang Luo, Jiaxing Zhang

**Affiliations:** 1 Department of Urology, The First Affiliated Hospital of Sun Yat-sen University, No. 58, Zhongshan road II, Guangzhou, 510080 People’s Republic of China; 2 Department of Emergency, The First Affiliated Hospital of Sun Yat-sen University, No. 58, Zhongshan road II, Guangzhou, 510080 People’s Republic of China; 3 Department of Oncology, The First Affiliated Hospital of Sun Yat-sen University, No. 58, Zhongshan road II, Guangzhou, 510080 People’s Republic of China; 4 Department of Oncology, The First Affiliated Hospital of Guangzhou University of Chinese Medicine, No. 16 Airport road, Guangzhou, 510405 People’s Republic of China

**Keywords:** circSDHC, E2F1 pathway, miR-127-3p, Renal cell carcinoma

## Abstract

**Background:**

There is increasing evidence that circular RNAs (circRNAs) have significant regulatory roles in cancer development and progression; however, the expression patterns and biological functions of circRNAs in renal cell carcinoma (RCC) remain largely elusive.

**Method:**

Bioinformatics methods were applied to screen for circRNAs differentially expressed in RCC. Analysis of online circRNAs microarray datasets and our own patient cohort indicated that circSDHC (hsa_circ_0015004) had a potential oncogenic role in RCC. Subsequently, circSDHC expression was measured in RCC tissues and cell lines by qPCR assay, and the prognostic value of circSDHC evaluated. Further, a series of functional in vitro and in vivo experiments were conducted to assess the effects of circSDHC on RCC proliferation and metastasis. RNA pull-down assay, luciferase reporter and fluorescent in situ hybridization assays were used to confirm the interactions between circSDHC, miR-127-3p and its target genes.

**Results:**

Clinically, high circSDHC expression was correlated with advanced TNM stage and poor survival in patients with RCC. Further, circSDHC promoted tumor cell proliferation and invasion, both in vivo and in vitro. Analysis of the mechanism underlying the effects of circSDHC in RCC demonstrated that it binds competitively to miR-127-3p and prevents its suppression of a downstream gene, *CDKN3*, and the E2F1 pathway, thereby leading to RCC malignant progression. Furthermore, knockdown of circSDHC caused decreased CDKN3 expression and E2F1 pathway inhibition, which could be rescued by treatment with an miR-127-3p inhibitor.

**Conclusion:**

Our data indicates, for the first time, an essential role for the circSDHC/miR-127-3p/CDKN3/E2F1 axis in RCC progression. Thus, circSDHC has potential to be a new therapeutic target in patients with RCC.

**Supplementary Information:**

The online version contains supplementary material available at 10.1186/s12943-021-01314-w.

## Background

Renal cell carcinoma (RCC) comprises approximately 3% of malignant tumors [1], and its incidence rate has been rising over the past decade. Around 90% of RCCs are the clear cell carcinoma (ccRCC) subtype [2]. Primary RCC is commonly treated with radical nephrectomy; however, despite surgical resection, approximately 30% of patients with RCC eventually develop metastasis, which is associated with high levels of mortality [3, 4]. During recent decades, a great variety of novel biomarkers and their underlying mechanisms have been discovered in RCC, and demonstrated significant relevance in clinical practice [5]. Nevertheless, the development and progression of RCC remain incompletely understood, and more efforts are required to identify the molecular mechanisms that promote RCC development and progression, to facilitate better treatment of this disease.

Circular RNAs (circRNAs) are a subclass of small RNAs characterized by being covalently closed loops, without a 5′ cap or 3′ poly-A tail [6, 7]. CircRNAs were initially considered to represent ‘noise’ generated during transcription, and to have no significant cellular functions [8]. Recently, due to the wide application of high throughput sequencing, numerous novel circRNAs have been discovered in mammalian cells [9]. Among these circRNAs, a large proportion have important roles in both physiological and pathological processes, including cancer development and progression [10]. Emerging evidence shows that there is an interactive relationship between micro RNAs (miRNAs) and circRNAs, referred to as the “miRNA sponge” effect [11]. During this process, circRNAs trap miRNAs on specific binding sites, thus preventing miRNA from interfering with mRNA expression. This process can mediate cancer progression [12]; for example, circMAPK4 acts as a sponge for miR-125a-3p, which participates glioma progression via the MAPK signaling pathway [13]. Further, circZNF609 can interact with and downregulate miR-138-5p, promoting RCC progression [14]; however, the biological functions and clinical significance of circRNAs in RCC remain largely unknown, and require elucidation.

Here, we conducted bioinformatics analysis of published circRNA microarray data from the Gene expression omnibus (GEO, https://www.ncbi.nlm.nih.gov/gds/) database and determined that circSDHC (hsa_circRNA_100372) may have an oncogenic role in RCC development and progression. By performing in vitro and in vivo experiments, we demonstrate that circSDHC serves as a sponge for miRNA-127-3p, thereby regulating the CDKN3/E2F1 axis. Therefore, circSDHC is a promising potential prognostic biomarker and therapeutic target in patients with RCC.

## Materials and methods

### Cell lines and cell culture

Human RCC cell lines (A498, 786-O, 769P and Caki-1), a human renal proximal tubular epithelial cell line (HK2), and a human embryonic kidney cell line (HEK-293 T), were purchased from the Chinese Academy of Sciences. 786-O, and 769P were cultured in RPMI 1640 (Gibco, China) supplemented with 10% FBS (PAN-Seratech, Germany). A498, Caki-1, HK2, and HEK-293 T were cultured in DMEM (Gibco, China) supplemented with 10% FBS (PAN-Seratech, Germany). The incubation environment was at 37 °C with 5% CO_2_. Cells were routinely checked for mycoplasma infection during cell culture (Beyotime, China).

### ccRCC patient samples and follow-up data

A total of 140 patients with ccRCC, who underwent radical nephrectomy without neoadjuvant chemotherapy or radiotherapy between 2002 and 2012, at the First Affiliated Hospital of Sun Yat-sen University (Guangzhou, China), were recruited into the study cohort. Patients were followed up regularly, with a median follow-up time of 99.0 months. Overall survival (OS) was defined as the duration from the date of surgery to the date of patient’s death for any reason. Formalin-fixed, paraffin-embedded (FFPE) samples of both tumor and adjacent normal tissue, were collected from these patients for analysis of RNA expression. Total RNA was extracted from tissue specimens using a nucleic acid isolation kit for FFPE (ThermoFisher, USA). Samples used in this study were approved by the Medical Ethics Committee of the First Affiliated Hospital of Sun Yat-sen University.

### Bioinformatics analysis

Microarray datasets were obtained from GEO by searching using the keywords “circRNA’” and “renal cell carcinoma”. Two datasets were retrieved: GSE100186, consisting of 4 tumors and matched adjacent normal tissue; and GSE137836, consisting of 3 primary tumors and 3 metastatic tumors. Arraystar Human circRNA microarray V2 chip info was downloaded for further analysis reference (https://www.ncbi.nlm.nih.gov/geo/query/acc.cgi?acc=GPl21825). The Cancer Genome Atlas (TCGA) clear cell renal cell carcinoma (ccRCC) sequencing and clinical data were downloaded from Firebrowse (http://firebrowse.org/). R (version 3.4.3) (https://www.r-project.org/) was used for subsequent data analysis.

### RNA and gDNA extraction

Total RNA samples were extracted from cells using Trizol (Invitrogen, USA) according to the manufacturer’s instructions. Genomic DNA (gDNA) was extracted using a genomic DNA isolation kit (Sangon Biotech, China).

### RNase R treatment, cDNA synthesis, and PCR

Aliquots of total RNA (2 μg) were incubated with or without 3 U/μg RNase R (Epicenter Technologies, USA) for 30 min at 37 °C and the product RNAs purified using an RNeasy MinElute cleaning Kit (Qiagen, Germany). Isolated RNA was first reverse-transcribed to cDNA using the PrimeScript RT Master Mix (Takara, China) containing random and oligo (dT) primers. Then, PCR was performed using GoTaq Green Master Mix (Promega, USA), according to the manufacturer’s instructions. Primer sequences are provided in Additional file 1: Table S1. PCR products were subjected to electrophoresis on a 2% agarose gels and visualized using Safe Green (Biosharp, China).

### Quantitative RT-PCR (qRT-PCR)

For qRT-PCR assays, 2X SYBR Green Pro Taq HS Premix II (AGbio, China) was used and reactions were conducted on QuantStudio 5 real-time-PCR instruments (ThermoFisher, USA). Primer sequences are provided in Additional file 1: Table S1. CircRNA and mRNA levels were normalized to those of *GAPDH*, while miRNA levels were normalized to those of small nuclear *U6*. The 2^−ΔΔCt^ method was used to calculate relative expression levels.

### Actinomycin D assay

Cells were cultured with or without 2 μg/ml actinomycin D (Sigma, USA) in medium. Then, the cells were harvested at different time points, followed by RNA extraction and qRT-PCR detection of RNA stability, as described above.

### Fluorescence in situ hybridization (FISH)

Cy3-labeled circSDHC and FAM-labeled miRNA-127-3p probes were synthesized by RiboBio (China). A Fluorescent in Situ Hybridization Kit (RiboBio, China) was used to hybridize the probes to cells. Images were captured on a confocal laser scanning microscope (FV1000; Olympus, Japan).

### Western blot

Cells were harvested and lysed on ice in RIPA buffer (ThermoFisher, USA) containing proteinase inhibitor (Beyotime, China). Then, lysates were incubated on ice for 15 min before being centrifuged for 15 min (13,000 RPM, 4 °C). Supernatants were collected and protein concentration measured using a BCA protein assay kit (ThermoFisher, USA). Protein samples (20 μg) were loaded in each lane of SDS-PAGE gels. After electrophoresis, proteins were transferred onto PVDF membranes, which were blocked in non-fat milk. Then, membranes were incubated overnight at 4 °C with primary antibody, and subsequently with secondary antibody for 1 h at room temperature. Hybridizations were detected using a western blot substrate kit (Tanon, China) on a FluorChem E System (ProteinSimple, USA). Antibodies used in western blots were as follows: CKDN3 (1:1000 dilution, Abcam, USA), E2F1 (1:1000 dilution, Cell signaling, USA), GAPDH (1:1000 dilution, Cell signaling, USA), CDK1 (1:1000 dilution, ThermoFisher, USA), CDK2 (1:1000 dilution, ThermoFisher, USA), HRP-conjugated goat anti-mouse (1:5000 dilution, Proteintech, China), and HRP-conjugated goat anti-rabbit antibody (15,000 dilution, Proteintech, China).

### Plasmid construction and siRNA interference assay

For circSDHC over-expression plasmids, human circSDHC cDNA was synthesized and cloned into a pLVX-cir vector (Genomeditech, China); empty vector was used as the negative control. For siRNA assays, two targeting siRNAs and one scrambled siRNA (negative control) were synthesized by RiboBio (China) (Additional file 1: Table S1). Both overexpression plasmid and siRNAs were transfected using Lipofectamine 3000 (Invitrogen, USA), according to the manufacturer’s instructions. Functional assays were carried out 48 h after transfection. Protein and RNA were harvested 48 h after transfection.

### Pull-down assay with biotinylated circSDHC

A biotinylated probe targeting the junction area of circSDHC was synthesized (RiboBio, China); an oligo probe served as the negative control. Briefly, to generate probe-coated beads, probes were incubated with streptavidin magnetic beads (Invitrogen, USA) at room temperature for 2 h. Then, the probe-coated beads were mixed with cell lysates overnight at 4 °C. After centrifuging to wash the beads, pulled down miRNAs were extracted using Trizol (Invitrogen, USA) and subjected to qRT-PCR.

### Pull-down assay with biotinylated miRNA

Biotinylated miR-127-3p and scrambled negative control miRNA were synthesized (RiboBio, China). Biotinylated miRNAs were transfected into cells using Lipofectamine 3000 (Invitrogen, USA), then cell lysates collected 48 h after transfection and incubated with streptavidin magnetic beads (Invitrogen, USA) at room temperature for 2 h. After centrifuging to wash the beads, pulled down circRNAs were extracted using Trizol (Invitrogen, USA) and subjected to qRT-PCR.

### Luciferase reporter assay

Twenty-four hours before transfection, 3 × 10^3^ HEK-293 T cells per well were seeded in 96-well plates. A mixture of 50 ng luciferase reporter vectors, 5 ng Renilla luciferase reporter vectors (pRL-TK), and different miRNA mimics were co-transfected into the cells. After 48 h of incubation, luciferase activity was measured using a dual luciferase reporter assay kit (Promega, USA) on a Varioskan LUX machine (Thermo, USA). Luciferase values were normalized to those of corresponding Renilla luciferase, and fold-changes in luciferase values calculated.

### Cell migration and invasion assays

Transwell assays were used to evaluate the invasion and migration ability of cells in vitro. Prior to assays, cells were starved by culture in serum-free medium for 8 h. Then, cells were collected, adjusted to a concentration of 1 × 10^5^ in 100 μl of serum-free medium, and added to transwell inserts (Corning, USA), which were coated with (in the invasion assay) and without (in the migration assay) 2% Matrigel (Corning, USA). Medium supplemented with 10% FBS was added to the lower chamber as a nutritional attractant. After incubation (8 h for migration assay and 16 h for invasion assay), transwell inserts were collected, fixed with 4% polyformaldehyde (Beyotime, China), and stained with 0.4% crystal violet (Beyotime, China) for 20 min. Cells on the upper surface were wiped out with a cotton swab, and invaded/migrated cells calculated by capturing five random fields under an Olympus IX83 inverted microscope (Japan).

### MTT assay

For MTT assays, 1 × 10^3^ cells were seeded in each well of a 96-well plate and incubated for a specific period of time. At the harvesting time point, 10 μl of 5 mg/ml MTT (Beyotime, China) was added to each well and incubated for 2 h. Then, the medium was aspirated and 100 μl of DMSO (MP Biomedicals, USA) added to each well, followed by brief shaking to dissolve the crystals, and measurement of absorbance at a wavelength of 490 nm.

### Hematoxylin and eosin (HE) and immunohistochemistry (IHC) staining

These procedures were performed as described previously [15, 16]. Antibodies used for IHC were as follows: CDKN3 (1:100 dilution, ThermoFisher, USA) and E2F1 (1:100 dilution, ThermoFisher, USA). Paraffin sections (thickness, 5 μm) were used for staining. Images were captured with an Olympus IX83 inverted microscope (Japan). Pathological samples were evaluated and scored separately by two qualified pathologists. The IHC scoring is as follows: 0 for no staining, 1+, 2+, 3+ and 4+ for 1–24, 25–49%, 50–74% and over 75% staining intensity, respectively.

### Animal experiments

All animal care and experimental procedures were conducted according to the guidelines of the National Institutes of Health, and were approved by the Institutional Animal Care and Use Committee of Sun Yat-sen University. BALB/c nude mice (3–4 weeks old) were purchased from Vital River Laboratory Animal Technology (China). Stable transfection of control vector and circSDHC shRNA was performed in the 786-O cell line, which was subsequently used for animal studies. For the metastasis experiment, there were eight mice per group. Cells (1 × 10^6^) transfected with control vector or shRNA were injected via tail veins. Mouse body weights were measured weekly. All mice were euthanized after 8 weeks and lung tissues collected and subjected to HE staining. Images were captured using an Olympus IX83 inverted microscope (Japan) and lung metastatic foci were counted in each sample. For the tumor growth study, eight mice were used in each group. Cells (5 × 10^6^) transfected with control vector or shRNA were inoculated subcutaneously into the left side of the body. Tumor size was measured weekly. All mice were euthanized after 4 weeks and subcutaneous tumors were dissected and collected. Final tumor weights were measured and tumor samples were subjected to HE and IHC staining.

### Statistical analysis

All statistical analyses were conducted using GraphPad Prism version 7.0 and R (version 3.4.3) (https://www.r-project.org/). The student’s t-test was used for comparisons between two experimental groups. Pearson coefficients were calculated to assess correlations. Survival analysis was performed using Kaplan–Meier curves, with application of the logrank test to calculate statistical significance. Univariate and multivariate hazard ratios (HRs) were calculated using Cox regression. All quantitative experimental data are from at least three repeated experiments and are presented as mean ± SD. All *p* values < 0.05 were considered significant (*P < 0.05; **P < 0.001; ***P < 0.0001).

## Results

### Oncogenic circRNA discovery and characterization of circSDHC in RCC

**Fig. 1:**
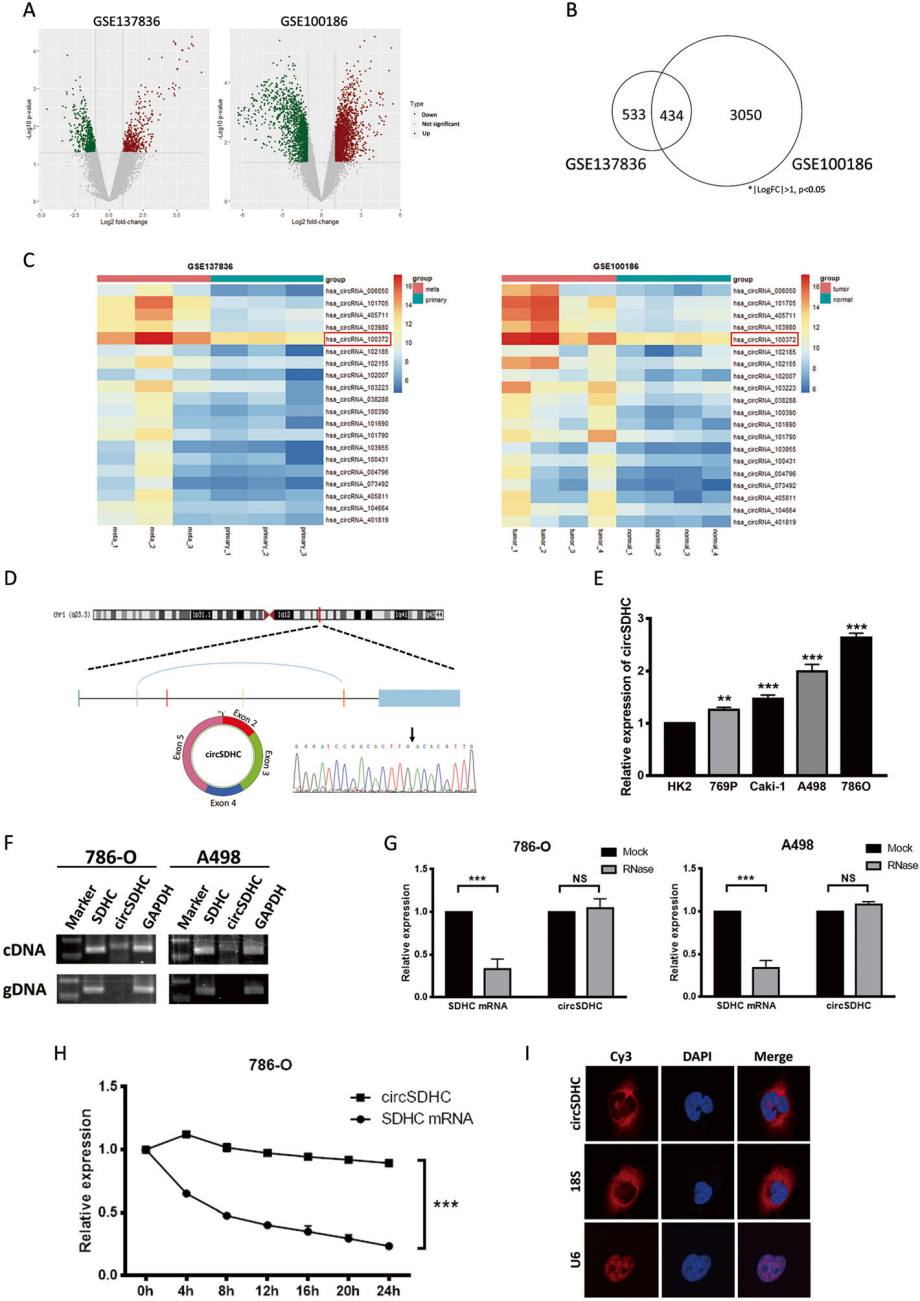
Oncogenic circRNAs discovery and characterization of circSDHC in RCC. **a**. Volcano plot of GSE137836 and GSE100186. Compared to the primary tumors (in GSE137836) and adjacent normal tissue (in GSE100186), red dots represent significantly up-regulated circRNAs, while green dots represent significantly down-regulated circRNAs. Grey dots represent circRNAs that are not significant. **b**. Venn plot of the two datasets. Common circRNAs with *p* < 0.05, |log2 (fold change)| > 1 are chosen. **c**. Heatmaps of the two datasets. The red color represents high expression whereas the blue color represents low expression. **d**. Schematic illustration of the circSDHC formation from SDHC gene in the chromosome 1. The back splicing junction was verified by Sanger sequencing. Black arrow indicates the specific junction. **e**. The expression level of circSDHC in normal kidney cell line HK2 and different RCC cell lines, measured by qRT-PCR. **f**. The existence of circSDHC was confirmed by RT-PCR and gel electrophoresis using convergent and divergent primers. circSDHC can only be amplified in cDNA. GAPDH serves as control. **g**. Stability of circSDHC and linear SDHC was assessed by RNase treatment followed by qRT-PCR. **h**. Stability of circSDHC and linear SDHC was assessed by Actinomycin D treatment followed by qRT-PCR at different time points. **i**. Cellular localization of circSDHC was detected by FISH. Nuclear was label with DAPI dye. The majority of circSDHC is within the cytoplasm. Data are mean ± SD, *n* = 3

Two GEO datasets (GSE137836 and GSE100186) from circRNA microarray chips analyzing human tissue samples were used to investigate the role of circRNA in RCC development and progression. The GSE137836 dataset was from three primary and three metastatic tumors, while GSE100186 includes data from four tumors and matched adjacent normal tissue. After analysis of differentially expressed genes, circRNAs with log2 fold-change > 1 or < − 1, and *p* < 0.05 were selected, illustrated in the volcano plot (Fig. 1a). Commonly altered circRNAs that overlapped across the two datasets, representing a consistent regulatory pattern in RCC, were extracted, yielding a subset of 434 circRNAs (Fig. 1b). To focus on circRNAs with the most oncogenic potential, those located on chromosomes regions frequently amplified in RCC were identified (Additional file 2: Table S2). Subsequently, the 20 most significantly up-regulated circRNAs (log2 fold-change > 2) were selected (Additional file 3: Table S3) and heatmaps of the expression of these circRNAs in the two datasets were plotted (Fig. 1c).

Next, the selected 20 most up-regulated circRNAs were validated in our patient cohort by evaluation of their expression patterns by qRT-PCR. The results for circSDHC which had higher expression in the tumor compared to adjacent normal tissues, were the most consistent across our cohort (Additional file 4: Fig. S1A), with up-regulation in patients who eventually developed metastasis (Additional file 4: Fig. S1B), and an association with poor patient overall survival (Additional file 4: Fig. S1C). Correlation analysis demonstrated that circSDHC expression was associated with clinicopathological parameter TNM stage (Table 1). Further, both univariate and multivariate Cox analysis indicated that higher circSDHC expression was associated with unfavorable survival outcomes (univariate HR: 3.3, 95% CI: 1.8–6.2; multivariate HR: 2.9, 95% CI: 1.5–5.6) (Table 2).

**Table 1:** Association of circSDHC expression with clinicopathological characteristics in 140 ccRCC patients

Parameter	Total	circSDHC expression		***p*** value
		High	Low	
**Age(y)**				
< 60	101	46	55	0.132
≥ 60	39	24	15	
**Gender**				
Female	42	23	19	0.580
Male	98	47	51	
**TNM stage**				
I	109	46	63	0.001
II-III	31	24	7	
**Fuhrman grade**				
1+ 2	88	39	49	0.115
3+ 4	52	31	21

**Table 2:** Univariate and multivariate Cox regression analyses of dfifferent parameters on overall survival

Parameter	Univariate Analysis		Multivariate Analysis	
	HR (95%CI)	***P*** Value	HR (95%CI)	***P*** Value
Age (≥ 60 vs. < 60 yr)	1.049 (1.020-1.078)	<0.001	1.041 (1.013-1.071)	0.004
Gender (Female vs. male)	1.692 (0.734-3.898)	0.217	–	–
TNM stage (II-III vs. I)	2.398 (1.477-3.894)	<0.001	1.405 (0.810-2.438)	0.226
Fuhrman (3+ 4 vs. 1+ 2)	2.074 (1.242-3.463)	0.005	1.689 (0.958-2.976)	0.070
Surgery type (Open vs. laparoscopy)	0.525 (0.251-1.100)	0.088	–	–
circSDHC expression (High vs. low)	3.324 (1.777-6.217)	<0.001	2.916 (1.508-5.637)	0.002
HR=hazard ratio. CI= confidence interval				

CircSDHC (Arraystar ID: hsa_circRNA_100372, circBase ID: hsa_circ_0015004) is derived from the *SDHC* gene on chromosome 1, resulting from back-splicing of exons 2, 3, 4, and 5 (385 bp). We conducted Sanger sequencing to confirm the back-splicing junctions of circSDHC (Fig. 1d). Compared with the normal kidney epithelial cell line HK2, four RCC cell lines (Caki-1, A498, 786-O, 769P) showed elevated circSDHC expression (Fig. 1e). PCR analysis confirmed that divergent primers could amplify the circSDHC from reverse-transcribed RNA (cDNA), but not from gDNA (Fig. 1f). Furthermore, circSDHC was more resistant to RNase R treatment, while *SDHC* mRNA was significantly degraded by this treatment (Fig. 1g). CircSDHC also had longer half-life than *SDHC* mRNA, as confirmed by actinomycin D treatment (Fig. 1h). In addition, the subcellular localization of circSDHC in 786-O cells was analyzed using a FISH assay, demonstrating that the majority of circSDHC localized to the cytoplasm (Fig. 1i).

### CircSDHC promotes RCC proliferation and aggression

**Fig. 2:**
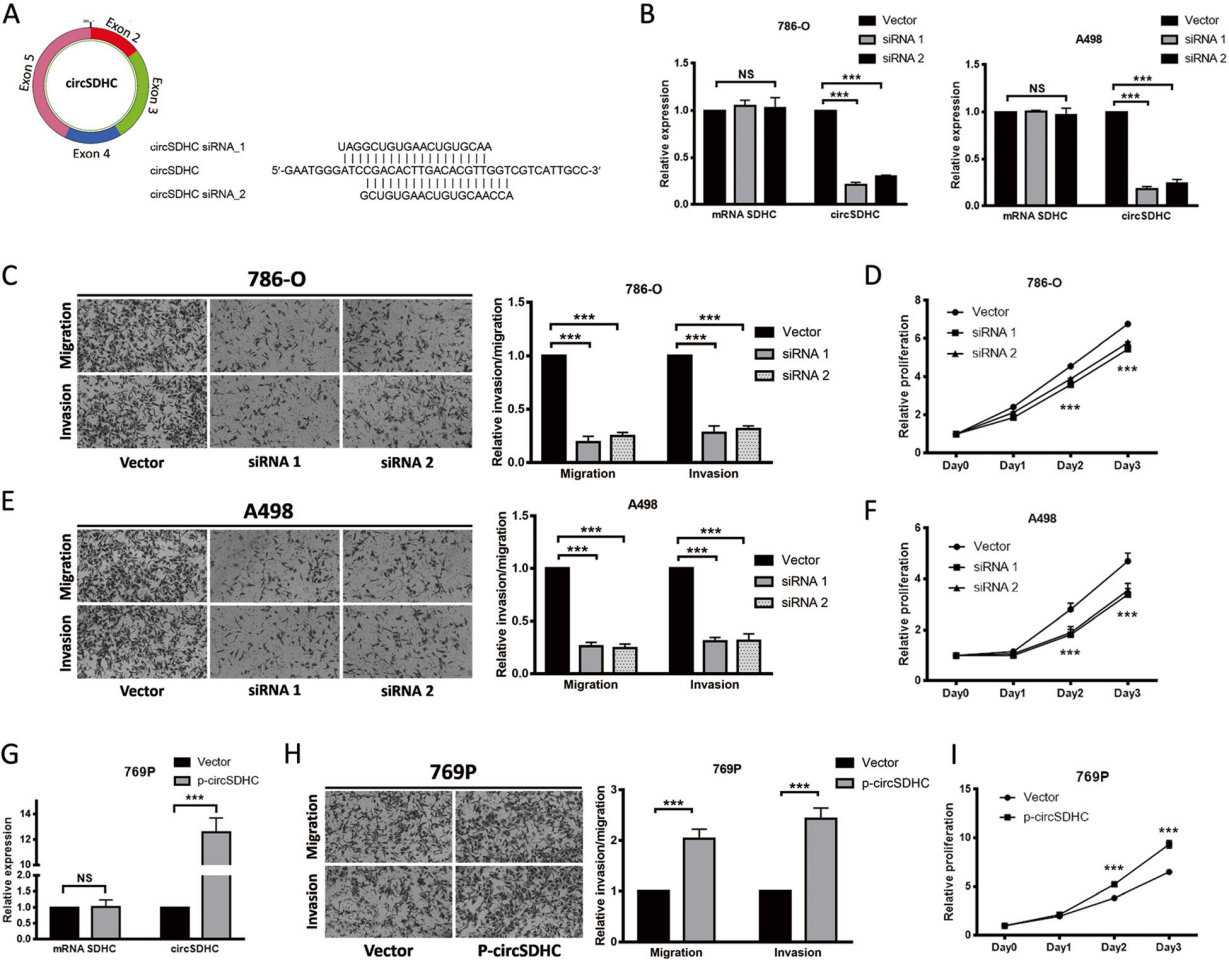
CircSDHC promotes the proliferation and aggressiveness of RCC. **a**. Target sites of siRNAs used in the knockdown experiment. Both siRNAs target back-splice junction of circPRMT5. **b**. The knockdown efficiency was measured by qRT-PCR in 786-O and A498 cell lines. **c** and **e**. Cell migration and invasion abilities of 786-O and A498 transfected with circSDHC siRNAs or control vector. Number of cells migrated or invaded was determined by counting the cells on five random microscopic field and calculating the mean. Level of migration and invasion were normalized to the control vector group. **d** and **f**. Cell proliferation ability of 786-O and A498 transfected with circSDHC siRNAs or control vector. The relative proliferative rate at different time points were normalized to day 0. **g**. The overexpression plasmid of circSDHC or control vector were transfected into 769P cell line, and expression level of circSDHC was measured by qRT-PCR. **h**. Cell migration and invasion abilities of 769P transfected with overexpression plasmid or control vector. **i**. Cell proliferation abilities of 769P transfected with overexpression plasmid or control vector. Data are mean ± SD, *n* = 3

To evaluate the biological roles of circSDHC in RCC, we conducted functional assays. To manipulate circSDHC expression, siRNAs targeting the back-splicing junction were constructed (Fig. 2a). Using the siRNAs, expression of circSDHC was successfully knocked down in two RCC cell lines (786-O and A498) with the highest circSDHC levels, without altering expression of the linear form of *SDHC* mRNA (Fig. 2b). Knockdown of circSDHC significantly decreased the migratory and invasive ability of 786-O and A498 cells (Fig. 2c, e). Further, proliferation of these cells was also suppressed on knockdown of circSDHC (Fig. 2d, f). To further investigate its function, a circSDHC overexpression vector was constructed, which significantly up-regulated circSDHC levels in another RCC cell line, 769P, which had relatively lower circSDHC abundance among the RCC cell lines (Fig. 2g). Consistent with findings from knockdown experiments, the migration, invasion, and proliferation rates of 769P cells were increased on overexpression of circSDHC (Fig. 2h, i). Additionally, we also tested the effect of circSDHC overexpression in normal kidney proximal tubular epithelial cell line HK2. The result showed that circSDHC could also promote malignancy in normal kidney cells, evidenced by increased growth, migration and invasion ability in HK2 after overexpression of circSDHC (Additional file 4: Fig. S1D-F).

### The tumor suppressor, miR-127-3p, is a target of circSDHC in RCC

**Fig. 3:**
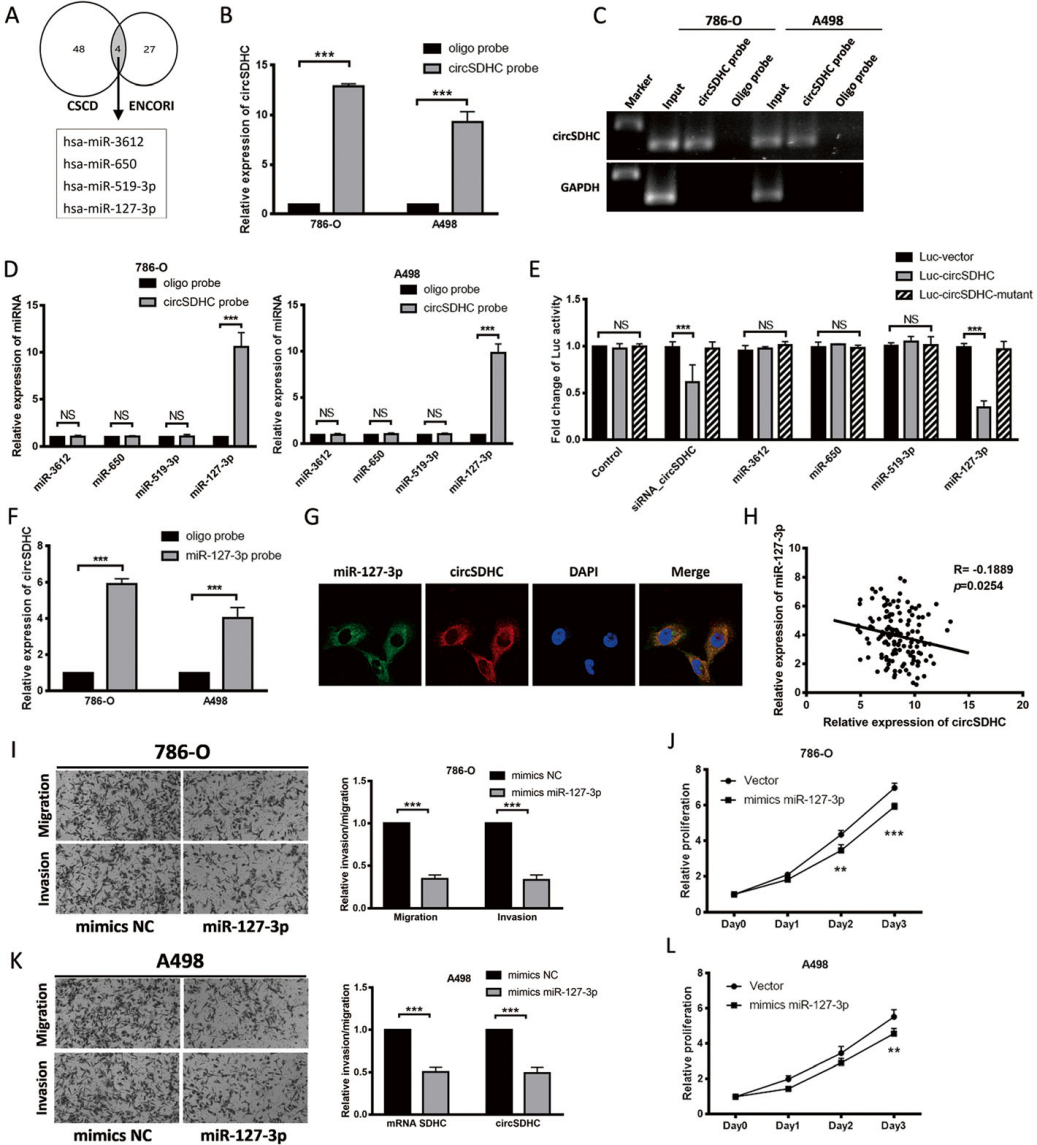
Tumor suppressor miR-127-3p is a target of circSDHC in RCC. **a**. Four miRNAs were predicted as the potential target of circSDHC in CSCD and ENCORI databases. **b** and **c**. Relative expression detected by qRT-PCR and gel electrophoresis of circSDHC in 786-O and A498 lysates after RNA pull down with circSDHC specific probe or oligo probe. Expression levels were normalized to oligo probe. GAPDH was used as negative control. **d**. Relative levels of candidate miRNAs were detected by qRT-PCR after being pull down by circSDHC probe or oligo probe. **e**. Luciferase reporter assay of 786-O with Luc-vector, Luc-circSDHC or Luc-circSDHC-mutant co-transfected with different candidate miRNAs. **f**. Relative level of circSDHC was detected by qRT-PCR after being pull down by miR-127-3p probe or oligo probe. **g**. Cellular localization of miR-127-3p (FAM) and circSDHC (Cy3) detected by FISH. Nuclear was label with DAPI dye. **h**. circSDHC expression was negatively correlative with miR-127-3p in our own patient cohort (*n* = 140). **i** and **k**. Cell migration and invasion abilities of 786-O and A498 transfected with mimics miR-127-3p or mimics NC. **j** and **l**. Cell proliferation ability of 786-O and A498 transfected with mimics miR-127-3p or mimics NC

A well-recognized function of circRNAs in cytoplasm is as sponges that regulate miRNA expression [17]. As our data indicated that circSDHC localizes to the cytoplasm, we attempted to determine whether it could function as a miRNA sponge. First, downstream miRNAs that may be regulated by circSDHC were predicted using two databases: the Cancer-specific circRNAs database (CSCD, http://gb.whu.edu.cn/CSCD/) and the Encyclopedia of RNA Interactomes (ENCORI, http://starbase.sysu.edu.cn/index.php). Four miRNAs, including miR-3612, miR-650, miR-519a-3p, and miR-127-3p, were identified as potential targets of circSDHC in both databases (Fig. 3a). To further evaluate the relationship between circSDHC and miRNAs, a biotin-labeled circSDHC probe was constructed, and qRT-PCR confirmed that the probe could specifically pull down the circSDHC, compared with a control oligo probe (Fig. 3b, c). Next, miRNAs binding to circSDHC in 786-O and A498 cells were pulled down using this probe. Subsequent qRT-PCR confirmed that miR-127-3p could bind to circSDHC in both 786-O and A498 cells (Fig. 3d). Next, we conducted luciferase reporter assays to validate the binding experiments, and the results confirmed the previous findings (Fig. 3e). Further, biotin-labeled miR-127-3p captured more circSDHC than a negative control probe (Fig. 3f) and FISH analysis in 786-O cells showed that circSDHC and miR-127-3p were co-localized in the cytoplasm (Fig. 3g). Furthermore, analysis of our ccRCC patient data showed a negative correlation between miR-127-3p and circSDHC (Fig. 3h). These results demonstrate that circSDHC can bind to miR-127-3p and act as a sponge for miR-127-3p.

According to TCGA ccRCC database, miR-127-3p expression is lower in tumor tissues of ccRCC, compared to normal tissue (Additional file 5: Fig. S2A). Moreover, it has previously been reported that lower expression of miR-127-3p correlates with early relapse in patients with RCC following nephrectomy [18]. These data suggested that miR-127-3p may have an inhibitory role in RCC development and progression. We also had similar observations in our patient cohort, where lower miR-127-3p expression was detected in tumor tissues than in adjacent normal adjacent tissues (Additional file 5: Fig. S2B). Further, patients who eventually developed distal metastasis had lower miR-127-3p expression in the tumor (Additional file 5: Fig. S2C). Moreover, lower miR-127-3p was correlated with unfavorable OS (Additional file 5: Fig. S2D). Consistent with these findings, overexpression of miR-127-3p in 786-O and A498 cells resulted in decreases in both aggression and proliferation (Fig. 3i-l).

### miR-127-3p inhibits RCC progression through downregulation of the CDKN3/E2F1 axis

**Fig. 4:**
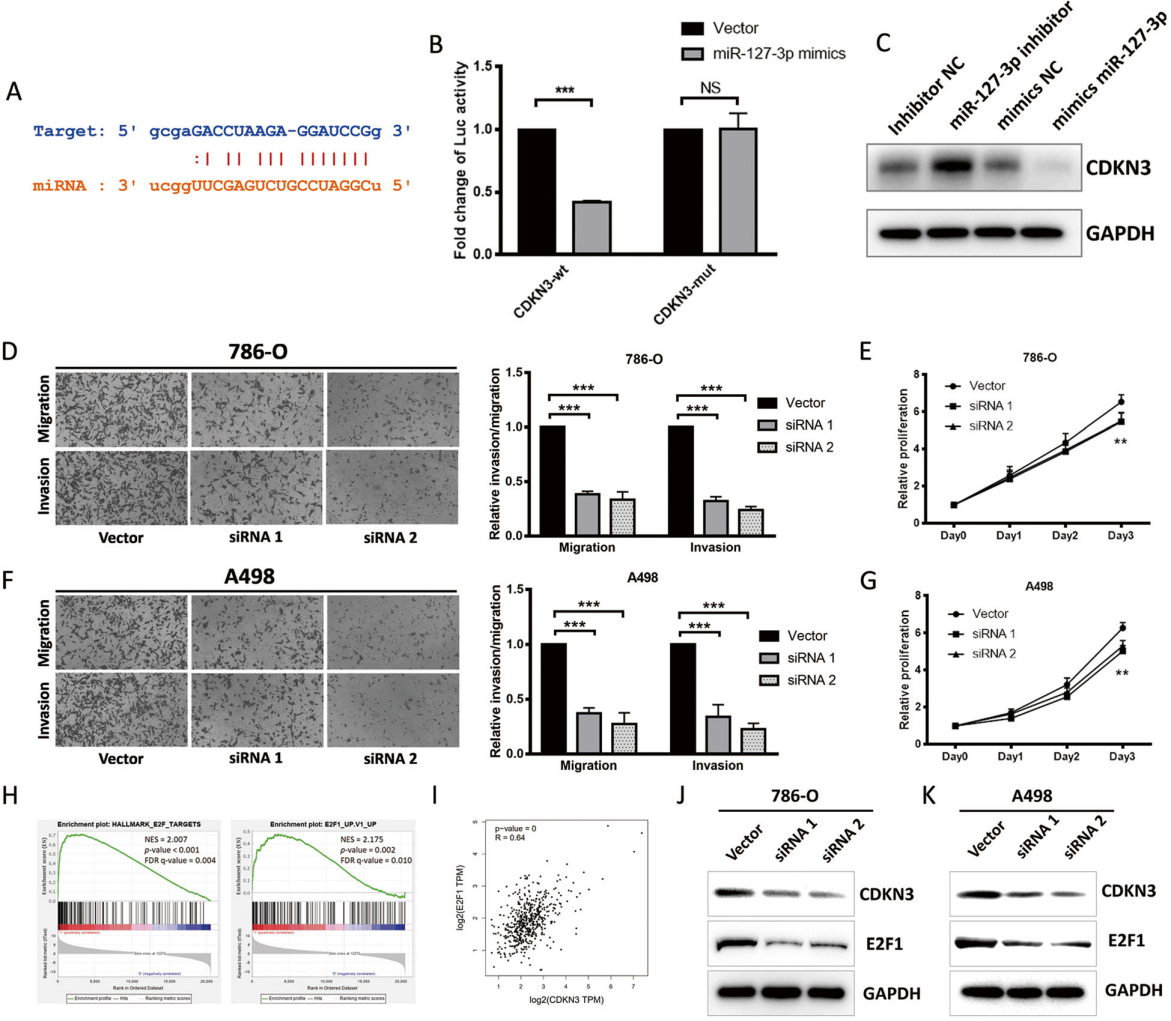
miR-127-3p inhibits RCC progression through downregulation of CDKN3/E2F1 axis. **a**. The binding site between miR-127-3p (orange) and CDKN3 (blue) predicted by ENCORI. **b**. Luciferase reporter assay of 786-O with Luc-CDKN3-wt or Luc-CDKN3-mut co-transfected with mimics miR-127-3p or mimics NC. **c**. Western blot of CDKN3 levels after 786-O cells were treated with miR-127-3p inhibitor, mimics miR-127-3p or negative control. **d** and **f**. Cell migration and invasion abilities of 786-O and A498 transfected with CDKN3 siRNAs or control vector. **e** and **g**. Cell proliferation ability of 786-O and A498 transfected with CDKN3 siRNAs or control vector. **h**. GSEA analysis of E2F1 pathway in TCGA patients with high and low CDKN3 expression (NES, normal enrichment score; FDR, false discovery rate). **i**. Correlation analysis between CDKN3 and E2F1 from GEPIA website (TCGA dataset, *n* = 523). **j** and **k**. Western blot of CDKN3 and E2F1 levels after 786-O and A498 cells were transfected with CDKN3 siRNAs or control vector

Next, the ENCORI database was used to determine target genes and downstream pathways regulated by miR-127-3p. Cyclin dependent kinase inhibitor 3 (CDKN3) was identified as a potential candidate gene regulated by miR-127-3p, with the binding classified as 7mer-m8 (Fig. 4a). Additionally, we conducted dual luciferase reporter assays, where vector containing wild-type CDKN3 sequence was co-transfected with miR-127-3p mimics, resulting in a reduction of luciferase activity by more than 50%. In contrast, transfection of a vector containing a mutant CDKN3 sequence had no impact on luciferase activity (Fig. 4b). Western blot assays confirmed this regulation (Fig. 4c). Moreover, in our patient cohort, we detected a negative relationship between miR-127-3p and CDKN3 expression levels (Additional file 6: Fig. S3A). These results indicate that miR-127-3p can inhibit RCC progression through downregulation of CDKN3.

According to the Gene Expression Profiling Interactive Analysis (GEPIA) database (http://gepia.cancer-pku.cn/index.html) [19], CDKN3 is expressed at higher levels in tumor samples than in normal tissue, and its levels were positively correlated with pathologic stage, and could predict unfavorable OS and disease-free survival outcomes in the ccRCC TCGA dataset (Additional file 6: Fig. S3B-E). CDKN3 knockdown in RCC cells led to a lower proliferation rate and diminished migration and invasion abilities (Fig. 4d-g). To predict the pathway involved downstream of CDKN3, we used Gene Set Enrichment Analysis (https://www.gsea-msigdb.org/gsea/index.jsp) [20, 21]. The results suggested E2F1 as a potentially involved pathway (Fig. 4h). E2F1 is a potent transcription regulator that participates in the development of many different types of cancer [22–25], and a previous paper reported that E2F1 is also involved in ccRCC progression [26]. GEPIA prediction indicated a positive correlation between CDKN3 and E2F1 levels (Fig. 4i) and western blot analysis following CDKN3 knockdown in 786-O and A498 cells confirmed this relationship (Fig. 4j-k).

### circSDHC regulates CDKN3/E2F1 and promotes RCC progression by acting as a sponge for miR-127-3p

**Fig. 5:**
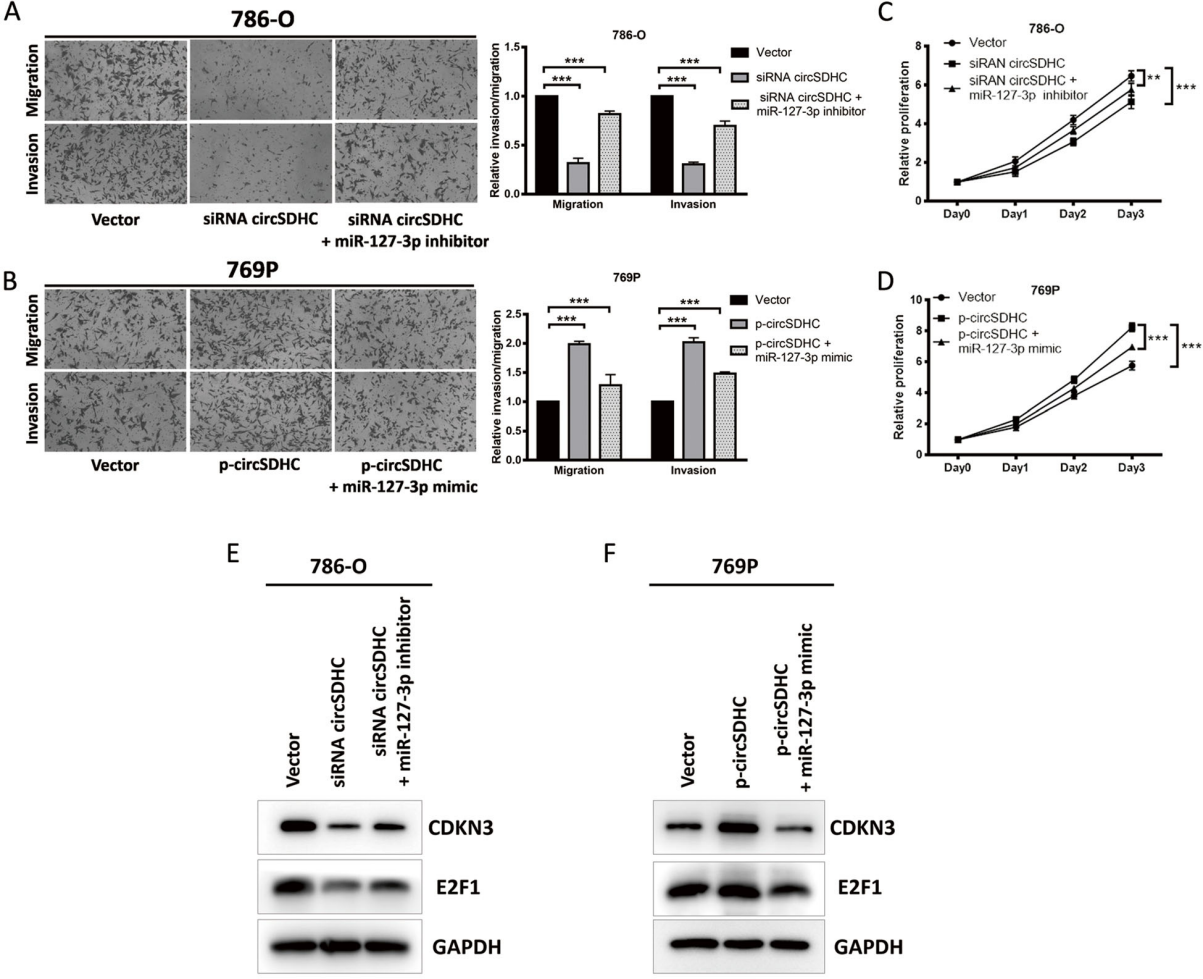
circSDHC regulates CDKN3/E2F1 and promotes RCC progression by the sponge effect towards miR-127-3p. **a**. Cell migration and invasion abilities of 786-O transfected with control vector, circSDHC siRNA alone or circSDHC siRNA plus miR-127-3p inhibitor. **b**. Cell migration and invasion abilities of 769P transfected with control vector, circSDHC overexpression plasmid alone or circSDHC overexpression plasmid plus miR-127-3p mimics. **c**. Cell proliferation ability of 786-O transfected with control vector, circSDHC siRNA alone or circSDHC siRNA plus miR-127-3p inhibitor. **d**. Cell proliferation ability of 769P transfected with control vector, circSDHC overexpression plasmid alone or circSDHC overexpression plasmid plus miR-127-3p mimics. **e**. Western blot of CDKN3 and E2F1 levels after 786-O transfected with control vector, circSDHC siRNA alone or circSDHC siRNA plus miR-127-3p inhibitor. **f**. Western blot of CDKN3 and E2F1 levels after 786-O transfected with control vector, circSDHC overexpression plasmid alone or circSDHC overexpression plasmid plus miR-127-3p mimics

To investigative whether circSDHC promotes RCC progression through its sponge effect on miR-127-3p, an miR-127-3p inhibitor and mimic were used to perform rescue experiments following circSDHC depletion or overexpression. Knockdown of circSDHC inhibited the aggression and proliferation of 786-O cells; however, the inhibition could be rescued by application of an miR-127-3p inhibitor (Fig. 5a, c). Similar effects were observed in 769P cells overexpressing circSDHC, which exhibited increased aggression and proliferation that could be attenuated by miR-127-3p mimic (Fig. 5b, d). Western blot assays also showed results consistent with the observed functional changes, where the CDKN3 and E2F1 pathway was less activated upon transfection of circSDHC siRNA, and could be rescued by administration of miR-127-3p inhibitor in 786-O cells (Fig. 5e). In contrast, the CDKN3/E2F1 pathway could be activated by transfection with the circSDHC overexpression vector and this effect was diminished by introduction of miR-127-3p mimic into 769P cells (Fig. 5f). Additionally, since CDKN3 is an important regulator of CDK1 and CDK2, we also tested the effect of circSDHC on CDK1 and CDK2. Our result proved that knockdown of circSDHC caused downregulation of CDKN3 and further decreased the expression of CDK1 and CDK2 (Additional file 6: Fig. S3F, G).

### Knockdown of circSDHC inhibits RCC tumor progression in vivo

**Fig. 6:**
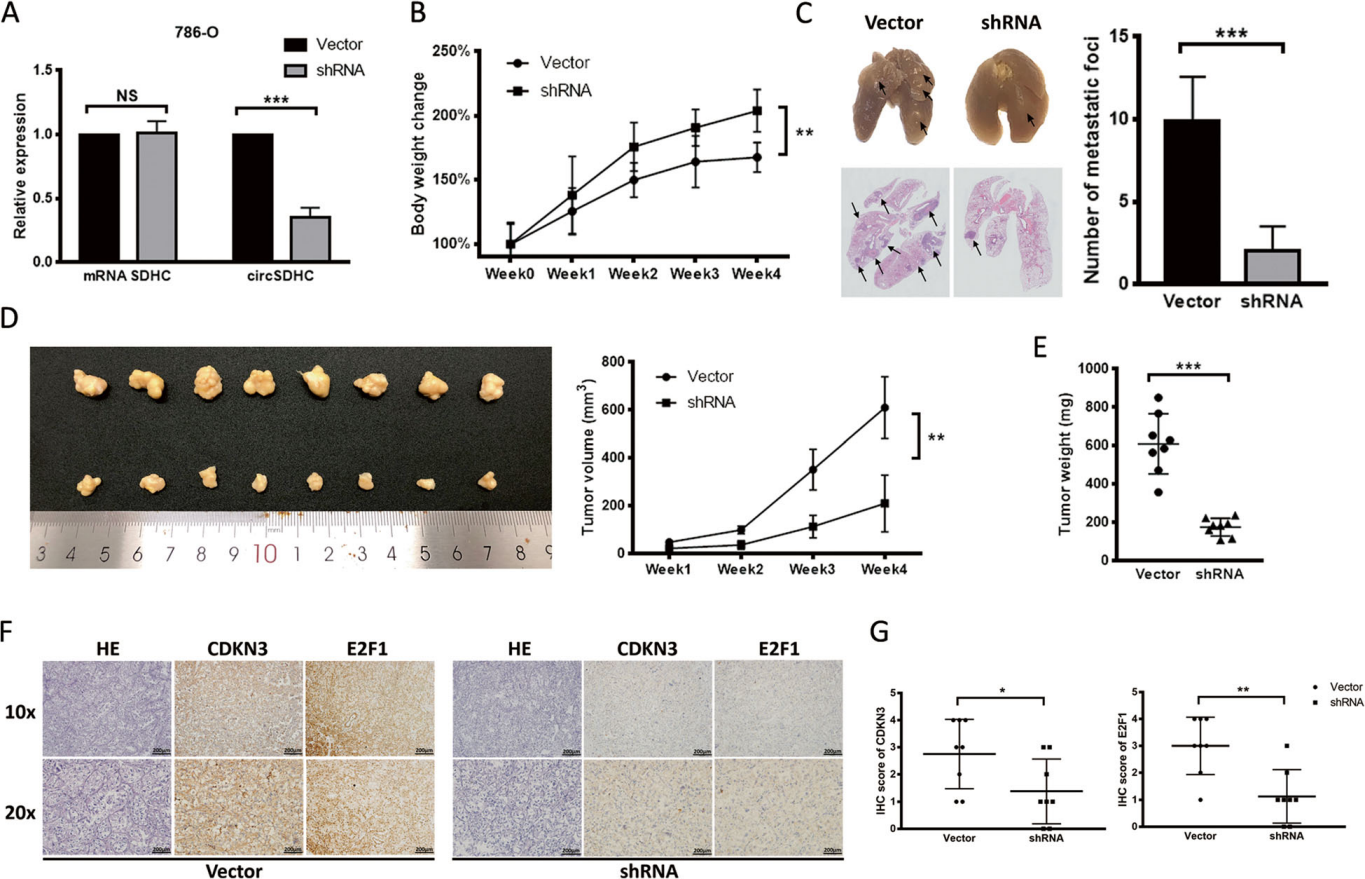
Knockdown of circSDHC inhibits the progression of RCC tumor in vivo. **a**. Stable circSDHC knockdown 786-O cell line establishment. Relative expression levels of linear SDHC and circSDHC were measured by qRT-PCR. Expression level were normalized to control vector group. **b**. Weekly mice body weight change in the metastasis experiment. 1 × 10^6^ stable circSDHC knockdown or control 786-O cells were injected via tail vein (*n* = 8 in each group). **c**. Representative images of gross and microscopic HE stain of the tumor-infiltrated lung (left). The metastatic foci in each mouse from two groups were counted under microscope and summarized (right). **d**. The picture of the gross tumors in dissected from subcutaneous xenograft model (left). 5 × 106 stable circSDHC knockdown or control 786-O cells were inoculated subcutaneously into the left side of the body. (*n* = 8 in each group). Weekly tumor volume change was recorded and showed (right). **e**. The final tumor weights in the subcutaneous xenograft model. **f**. HE and IHC staining of the tumors from subcutaneous xenograft model. **g**. Summary and statistical analysis of CDKN3 and E2F1 IHC scores

To evaluate the oncogenic effect of circSDHC in vivo, 786-O cells were stably transfected with an shRNA targeting circSDHC, while cells transfected with a scrambled vector were used as a negative control (Fig. 6a). Mice injected with circSDHC knockdown cancer cells demonstrated less cachexia than the control group, as represented by changes in mouse body weight (Fig. 6b). After 8 weeks, mice in the experimental and control groups were euthanized and lung tissues collected. On both gross and microscope examination, lungs from mice injected with circSDHC knockdown cancer cells had less metastatic foci than controls (Fig. 6c). Further, evaluation of subcutaneous tumor formation demonstrated that the circSDHC knockdown group had significantly lower overall mean tumor volume (Fig. 6d) and tumor weight (Fig. 6e) than the control group. IHC analysis of these subcutaneous tumors revealed that CDKN3 and E2F1 expression levels were decreased in the circSDHC knockdown group (Fig. 6f), and the IHC scores of CDKN3 and E2F1 were significant between two groups (Fig. 6g). These results demonstrate that knockdown of circSDHC inhibits RCC tumor progression in vivo.

## Discussion

circRNAs were once considered noise generated by transcription, with no significant biological function [8]; however, the development of high-throughput sequencing has revealed that there are actually a great variety of functional circRNAs in mammalian cells [9]. CircRNAs have unique functions in regulation of gene expression [9], and can influence the development and progression of many different types of cancer [13, 27–32]. Increasing numbers of circRNAs are being identified as potentially promising biomarkers [5]; however, the functions of circRNAs in RCC remain largely unknown, and warrant further exploration.

In our study, we first acquired circRNA microarray data from the GEO database: one dataset comparing tumors and adjacent normal tissue, and the other one compared primary tumors with matched metastatic lesions. Using filtering steps, we identified a number of circRNAs located on chromosomes that are frequently amplified in ccRCC, as having substantial oncogenic potential. After testing in our own patient cohort, circSDHC emerged as the most consistently associated circRNA, exhibiting higher expression in ccRCC tissue and correlation with unfavorable outcomes. In mechanistic experiments, circSDHC was found to downregulate mir-127-3p expression via a sponge effect, thereby activating the CDKN3/E2F1 pathway, and promoting RCC cell proliferation and metastasis. Moreover, in vivo animal studies confirmed the oncogenic characteristics of circSDHC. To our best knowledge, this is the first report on the expression and regulatory function of circSDHC in RCC.

There is accumulating evidence supporting the role of circRNAs as sponges for miRNAs in mediating various biological functions [17]. Notably, previous studies have demonstrated that circRNA cytoplasmic localization is closely associated with miRNA sponge effects. In the present study, we confirmed that circSDHC was predominantly distributed in the cytoplasm by FISH analysis and used two databases (CSCD and ENCORI) to predict miRNAs potentially bound by circSDHC. Subsequently, we used a biotin-labeled probe targeting circSDHC and luciferase reporter assays to confirm that miR-127-3p is a target of circSDHC. Further, analysis of TCGA dataset and data from our own patient cohort showed lower expression of miR-127-3p in tumor, compared with normal tissues. Our findings are consistent with previous studies, which proved that miR-127-3p functions as a tumor suppressor in several different cancers, including osteosarcoma [33], oral squamous cell carcinoma [34], and prostate cancer [35].

We also identified CDKN3 and its downstream E2F1 pathway as a target of miR-127-3p. CDKN3, as a CDK1 and CDK2 inhibitor protein, is traditionally considered a negative regulator of cell cycle progression [36]. Despite the negative regulatory effects of CDKN3 on CDK1 and CDK2, an oncogenic role for aberrant overexpression of CDKN3 has been implicated in numerous types of human cancer, including prostate cancer [37], gastric cancer [38], nasopharyngeal carcinoma [39], and esophageal cancer [40]. In esophageal cancer, CDKN3 influences cancer progression by promoting the cell cycle and chemo-resistance [40]. Moreover, CDKN3 can act as a positive regulator of CDK and Cyclin in certain cancer type, including gastric cancer, ovarian cancer and esophageal cancer [38, 41, 42]. Our data showed similar result, in which CDKN3 can upregulate the expression of CDK1 and CDK2. Further, the downstream E2F1 pathway is well-established as oncogenic, as it promotes the growth and metastasis of multiple cancer types [22–25]. Specifically, E2F1 promotes tumor malignancy and correlates with TNM stage in ccRCC [26]. Therefore, we predicted that the CDKN3/E2F1 pathway may be a target of circSDHC and miR-127-3p. According to the results of bioinformatics analysis, dual luciferase reporter assays, and rescue experiments, we verified a novel regulatory axis comprising circSDHC/miR-127-3p/CDKN3/E2F1 in RCC.

## Conclusion

In summary, our study demonstrates that circSDHC is overexpressed in ccRCC and that its overexpression is correlated with inferior survival. Further, circSDHC promotes ccRCC progression and metastasis by acting as a sponge for miR-127-3p, which is a tumor suppressor that downregulates the activity of CDKN3/E2F1 axis. These results suggest that circSDHC is a potential novel biomarker and therapeutic target in ccRCC.

## Supplementary Information

**Additional file 1: Table S1.** Primers and DNA/RNA sequences used in this study. (XLS 25 kb)

**Additional file 2: Table S2.** Frequently amplified chromosomes in RCC. (XLS 23 kb)

**Additional file 3: Table S3.** Most significant up-regulated circRNAs in amplificated chromosomes.

**Additional file 4: Figure S1.** CircSDHC expression and clinical parameters correlation in our own patient cohort, and the effect of circSDHC on HK2 cell. **A**. Relative expression of circSDHC between tumors and adjacent normal tissues in our own patient cohort (*n* = 140). **B**. Relative expression of circSDHC in tumor samples of patients who eventually developed metastasis compared to those didn’t develop metastasis (n = 140). **C**. Kaplan–Meier curve of OS between patients with high and low circSDHC expression (n = 140). Median circSDHC expression was used as the cut-off value. Log-rank test was used to calculate the *p* value. **D**. The overexpression plasmid of circSDHC or control vector were transfected into HK2 cell line, and expression level of circSDHC was measured by qRT-PCR. **E**. Cell proliferation abilities of HK2 transfected with overexpression plasmid or control vector. Data are mean ± SD, *n* = 3. **F**. Cell migration and invasion abilities of HK2 transfected with overexpression plasmid or control vector.

**Additional file 5: Figure S2.** Mir-127-3p expression and clinical parameters correlation in TCGA dataset and our own patient cohort. **A**. Relative expression of miR-127-3p between tumors and adjacent normal tissues in TCGA dataset (*n* = 255). **B**. Relative expression of circSDHC between tumors and adjacent normal tissues in our own patient cohort (n = 140). **C**. Relative expression of miR-127-3p in tumor samples of patients who eventually developed metastasis compared to those didn’t develop metastasis (n = 140). **D**. Kaplan–Meier curve of OS between patients with high and low miR-127-3p expression (n = 140). Median miR-127-3p expression was used as the cut-off value. Log-rank test was used to calculate the p value.

**Additional file 6: Figure S3.** *CDKN3* expression and clinical parameters correlation in TCGA dataset and our own patient cohort. **A**. miR-127-3p expression was negatively correlative with CDKN3 in our own patient cohort (*n* = 140). **B**. Relative expression analysis of CDKN3 between tumors and adjacent normal tissues from GEPIA website (TCGA dataset, *n* = 523). **C**. Relative expression analysis of CDKN3 among different clinical stage from GEPIA website (TCGA dataset, n = 523). **D** and **E**. Kaplan–Meier curve of OS and Disease free survival (DFS) between patients with high and low CDKN3 expression from GEPIA website (TCGA dataset, *n* = 516). Median CDKN3 expression was used as the cut-off value. Log-rank test was used to calculate the *p* value. **F** and **G**. Western blot of CDKN3, CDK1 and CDK2 levels after 786-O and A498 cells were transfected with circSDHC siRNAs or control vector.

## Data Availability

For all data requests, please contact the corresponding author.

## Abbreviations

CSCD: Cancer-specific circRNAs database
circRNAs: Circular RNAs
miRNAs: Micro RNAs
ENCORI: Encyclopedia of RNA Interactomes
FFPE: Formalin-fixed paraffin-embedded
FISH: Fluorescence in situ hybridization
GEO: Gene expression omnibus
GEPIA: Gene Expression Profiling Interactive Analysis
gDNA: Genomic DNA
HR: Hazard ratio
HE: Hematoxylin and eosin
IHC: Immunohistochemistry
OS: Overall survival
qRT-PCR: Quantitative RT-PCR
RCC: Renal cell carcinoma
ccRCC: Clear cell carcinoma
TCGA: The Cancer Genome Atlas
