# Supplementary figures and images for: Circular RNA circSDHC serves as a sponge for miR-127-3p to promote the proliferation and metastasis of renal cell carcinoma via the CDKN3/E2F1 axis
Source: Mol Cancer. 2021 Jan 20;20:19. doi: 10.1186/s12943-021-01314-w (PMC7816303; doi:10.1186/s12943-021-01314-w)

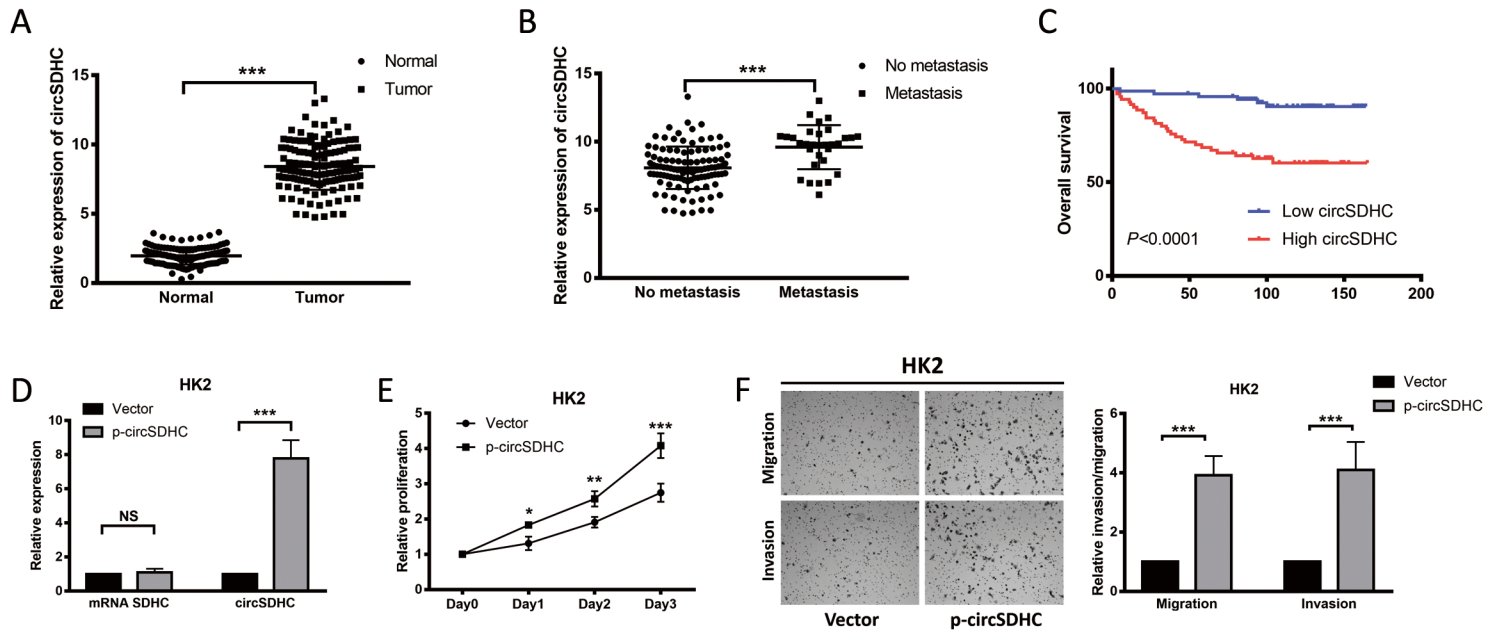

Supplement: Supplementary file 4 — Additional file 4: Figure S1. CircSDHC expression and clinical parameters correlation in our own patient cohort, and the effect of circSDHC on HK2 cell. A. Relative expression of circSDHC between tumors and adjacent normal tissues in our own patient cohort (n = 140). B. Relative expression of circSDHC in tumor samples of patients who eventually developed metastasis compared to those didn’t develop metastasis (n = 140). C. Kaplan–Meier curve of OS between patients with high and low circSDHC expression (n = 140). Median circSDHC expression was used as the cut-off value. Log-rank test was used to calculate the p value. D. The overexpression plasmid of circSDHC or control vector were transfected into HK2 cell line, and expression level of circSDHC was measured by qRT-PCR. E. Cell proliferation abilities of HK2 transfected with overexpression plasmid or control vector. Data are mean ± SD, n = 3. F. Cell migration and invasion abilities of HK2 transfected with overexpression plasmid or control vector. [file 12943_2021_1314_MOESM4_ESM.pdf]

A

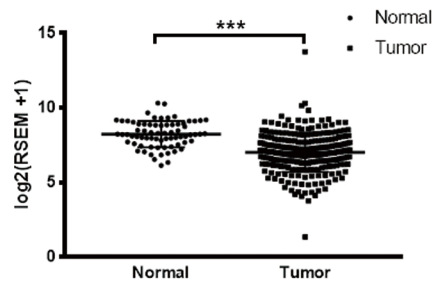

B

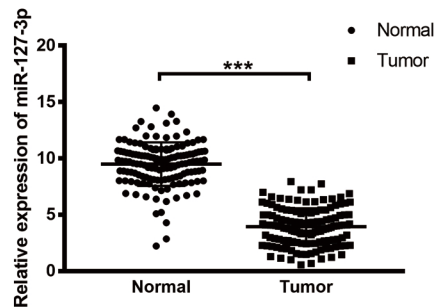

C

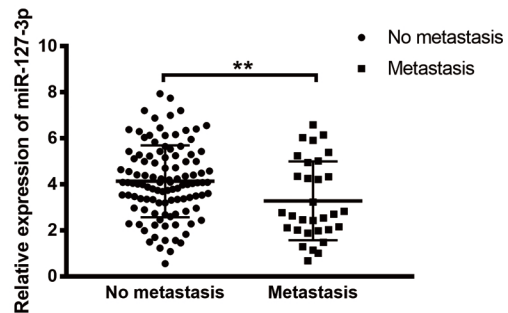

D

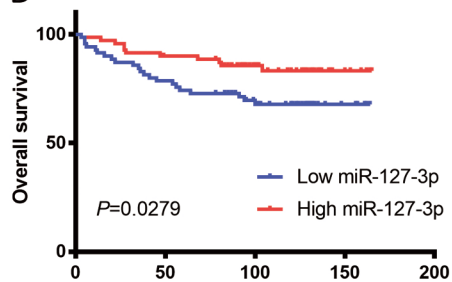

Supplement: Supplementary file 5 — Additional file 5: Figure S2. Mir-127-3p expression and clinical parameters correlation in TCGA dataset and our own patient cohort. A. Relative expression of miR-127-3p between tumors and adjacent normal tissues in TCGA dataset (n = 255). B. Relative expression of circSDHC between tumors and adjacent normal tissues in our own patient cohort (n = 140). C. Relative expression of miR-127-3p in tumor samples of patients who eventually developed metastasis compared to those didn’t develop metastasis (n = 140). D. Kaplan–Meier curve of OS between patients with high and low miR-127-3p expression (n = 140). Median miR-127-3p expression was used as the cut-off value. Log-rank test was used to calculate the p value. [file 12943_2021_1314_MOESM5_ESM.pdf]

**A**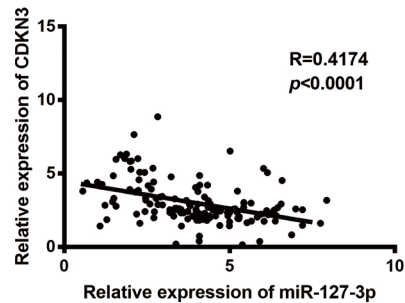**B**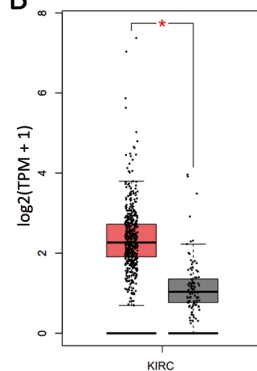**C**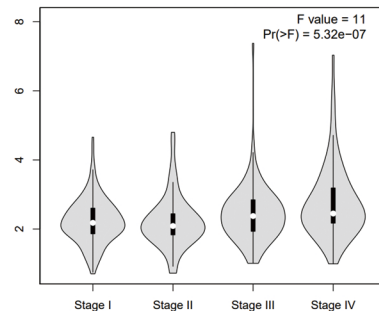**D**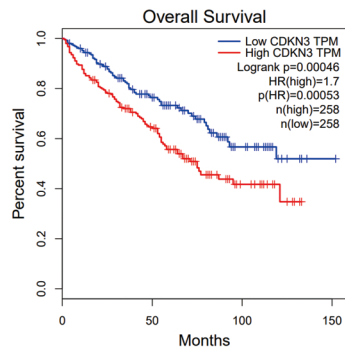**E**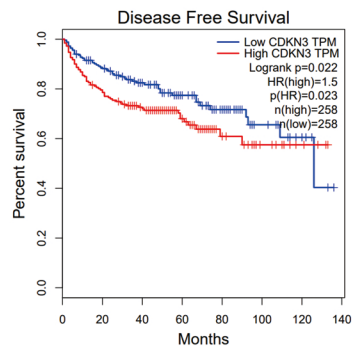**F**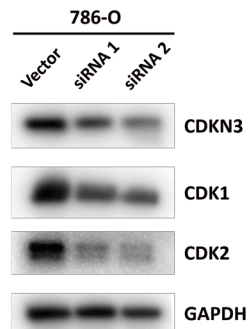**G**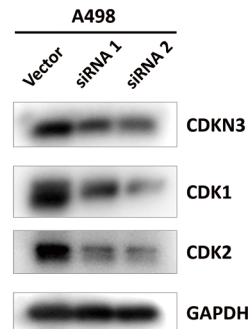

Supplement: Supplementary file 6 — Additional file 6: Figure S3. CDKN3 expression and clinical parameters correlation in TCGA dataset and our own patient cohort. A. miR-127-3p expression was negatively correlative with CDKN3 in our own patient cohort (n = 140). B. Relative expression analysis of CDKN3 between tumors and adjacent normal tissues from GEPIA website (TCGA dataset, n = 523). C. Relative expression analysis of CDKN3 among different clinical stage from GEPIA website (TCGA dataset, n = 523). D and E. Kaplan–Meier curve of OS and Disease free survival (DFS) between patients with high and low CDKN3 expression from GEPIA website (TCGA dataset, n = 516). Median CDKN3 expression was used as the cut-off value. Log-rank test was used to calculate the p value. F and G. Western blot of CDKN3, CDK1 and CDK2 levels after 786-O and A498 cells were transfected with circSDHC siRNAs or control vector. [file 12943_2021_1314_MOESM6_ESM.pdf]
